# Globulin, the albumin-to-globulin ratio, and fibrinogen perform well in the diagnosis of Periprosthetic joint infection

**DOI:** 10.1186/s12891-021-04463-7

**Published:** 2021-06-25

**Authors:** Huhu Wang, Haikang Zhou, Rendong Jiang, Zhenhao Qian, Fei Wang, Li Cao

**Affiliations:** Department of Orthopaedics, First Affiliated Hospital of Xinjiang Medical University, Urumqi, Xinjiang, China

**Keywords:** Periprosthetic joint infection, Diagnosis, Sensitivity, Specificity, Globulin, Albumin-to-globulin ratio, Fibrinogen

## Abstract

**Background:**

Although periprosthetic joint infection (PJI) is a severe complication of total joint arthroplasty (TJA), the diagnosis of PJI remains challenging. Albumin (ALB), globulin (GLB), the albumin-to-globulin ratio (AGR), and fibrinogen could be indicators of the body’s inflammatory state. This study aimed to compare the diagnostic accuracy of these biomarkers with that of other inflammatory biomarkers in PJI patients.

**Methods:**

We conducted a retrospective cohort study that included a consecutive series of patients undergoing debridement antibiotic irrigation and implant retention (DAIR), one-stage or the first stage of a two-stage revision total knee arthroplasty (TKA) or total hip arthroplasty (THA) for acute (*n* = 31) or chronic (*n* = 51) PJI, or revision TKA or THA for aseptic failures (*n* = 139) between January 2017 and December 2019 in our hospital. The 2013 criteria of the Musculoskeletal Infection Society (2013 MSIS) were used as the reference standard for the diagnosis of PJI. The preoperative ALB, GLB, AGR, fibrinogen, D-dimer, platelet count, fibrin degradation product (FDP), platelet-to-lymphocyte (PLR), platelet count to mean platelet volume ratio (PVR), neutrophil-to-lymphocyte ratio (NLR), erythrocyte sedimentation rate (ESR) and C-reactive protein (CRP) levels were assessed. The receiver operating characteristic curve (ROC), sensitivity, and specificity were utilized to compare different biomarkers.

**Results:**

Compared with the aseptic patients, the GLB, D-dimer, fibrinogen, FDP, platelet count, PVR, PLR, NLR, ESR, and CRP levels of PJI patients were significantly higher (*P* < 0.01); however, the ALB and AGR levels were significantly lower (*P* < 0.01). The area under the curve (AUC), sensitivity and specificity were 0.774, 67.50, 77.54% for ALB; 0.820, 57.50, 89.86% for GLB; 0.845, 66.25, 93.48% for AGR; 0.832, 78.48, 78.95% for fibrinogen; 0.877, 81.48, 85.07% for ESR; 0.909, 83.95, 88.89% for CRP; 0.683, 55.22, 75.83% for D-dimer; 0.664, 38.81, 88.33% for FDP; 0.678, 52.44, 79.86% for platelet count; 0.707, 48.78, 86.33% for PVR; 0.700, 51.22, 80.58% for PLR; and 0.678, 52.44, 81.30% for NLR, respectively. In the clinic, GLB, AGR and fibrinogen could be used for diagnosis of patients suspected of having PJI.

**Conclusion:**

Our study demonstrated that GLB, AGR, and fibrinogen were promising biomarkers in the diagnosis of PJI.

## Background

Periprosthetic joint infection (PJI), a severe complication of total hip arthroplasty (THA) or total knee arthroplasty (TKA), is one of the major causes of joint revision surgery [1–4]. Furthermore, it has been reported that the incidence of PJI ranges between 2.0 and 2.4% for THA and TKA [5]. A timely and accurate diagnosis of PJI is essential to preserve the implanted prosthesis, restore adequate joint function, and even reduce the morbidity rate [6, 7]. However, the diagnosis of PJI remains a challenge due to the lack of a “gold standard” and the fact that no currently available test is capable of absolute accuracy [8, 9].

The criteria provided by the Musculoskeletal Infection Society (MSIS) in 2011, modified in 2013, have been widely used in clinics [10, 11]. Although the diagnostic criteria introduced by MSIS have undoubtedly dramatically improved the diagnosis of PJI, some individuals still fail to be diagnosed even after surgery [12]. Therefore, numerous diagnostic tests for new biomarkers and new molecular techniques have been evaluated [7, 13–18]. Among these new biomarkers, blood biomarkers may be attractive due to their convenience, especially for some routing tests administered to all inpatients [4, 18].

Albumin (ALB), one of the main components of serum proteins, is negatively interrelated with the inflammatory process [19, 20]. Hypoalbuminemia, an index of malnutrition historically, has recently been a biomarker of inflammation [21, 22]. Serum globulin (GLB), such as components of complements and ceruloplasmin, increases during the inflammatory process [23, 24]. Therefore, the albumin-to-globulin ratio (AGR), taking ALB and GLB into account, is a promising biomarker for inflammation [25, 26], but its application in detecting PJI has not been studied. D-dimer, fibrinogen, fibrin degradation product (FDP), and platelet count are new biomarkers of PJI, and they perform well in many studies [8, 27]. Parvizi et al. [18] and Young-Min Kwon et al. [28] found that the platelet count to the mean platelet volume ratio (PVR) had specificity for PJI and could be used together with other biomarkers to improve the diagnostic performance of PJI. The neutrophil-to-lymphocyte ratio (NLR) and the platelet-to-lymphocyte ratio (PLR) are simple biomarkers of inflammation in many diseases, such as hepatitis virus infection, rheumatic diseases, and infective endocarditis [29–31].

We performed this study to verify the PJI diagnostic accuracy of ALB, GLB, AGR, and fibrinogen. The assumption was that ALB, GLB, AGR, and fibrinogen would be promising biomarkers for PJI diagnosis. We compared these new biomarkers with other inflammatory biomarkers, namely, D-dimer, FDP, platelet count, PVR, PLR, NLR, erythrocyte sedimentation rate (ESR) and C-reactive protein (CRP).

## Methods

### Study design and sample size estimation

**Fig. 1:**
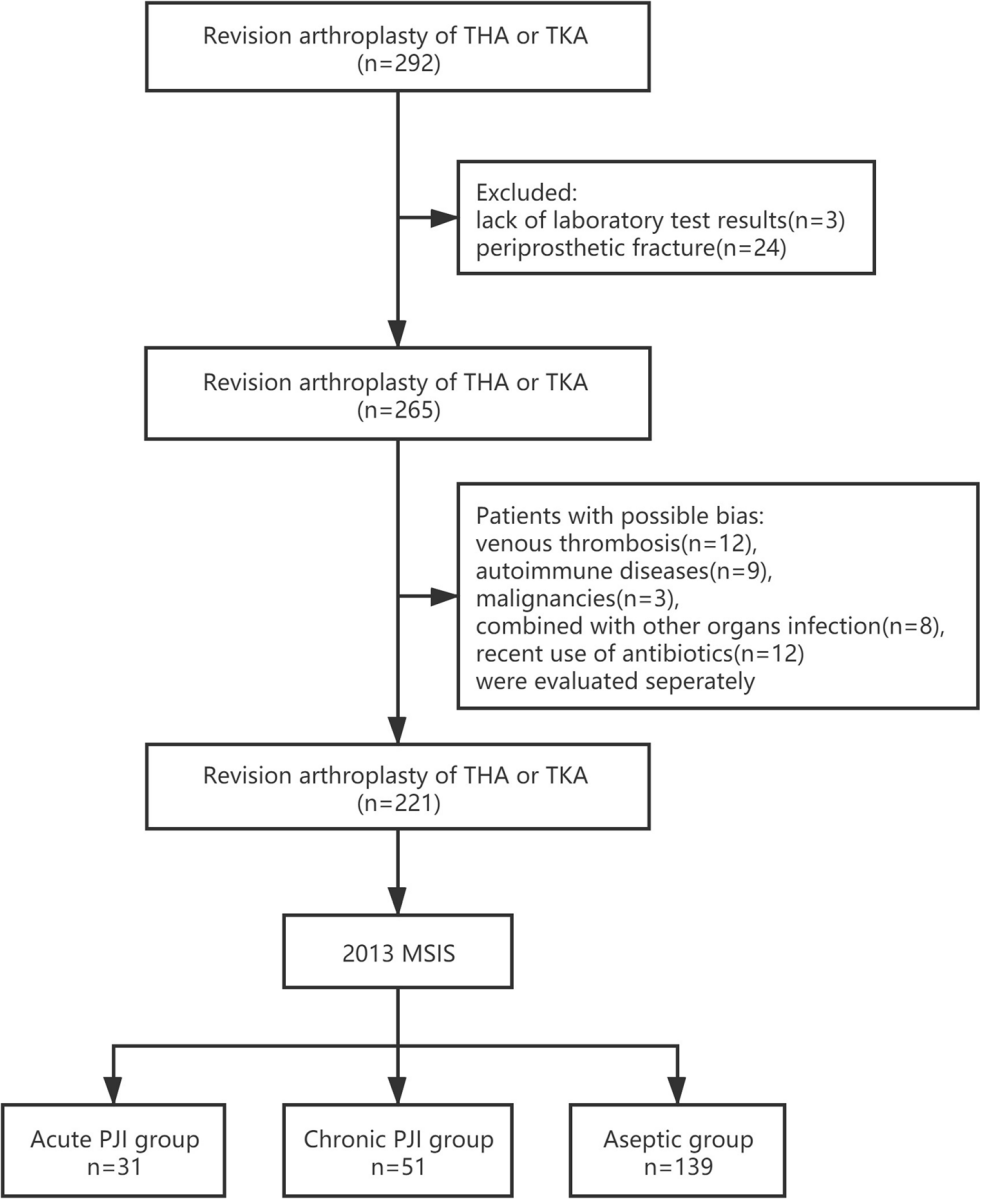
Flowchart of the included and excluded patients in this study. PJI, periprosthetic joint infection; TKA, total knee arthroplasty; THA, total hip arthroplasty

The sample size was calculated using PASS 11 software (NCSS). The incidence rate of PJI in TKA and THA revision surgery was estimated at 30%. An area under the receiver operating characteristic (ROC) curve (AUC) of 0.75 was set for AUC 1, and an AUC of 0.6 was set for AUC 0 (floor level). A one-sided test was utilized to obtain a power (1-beta) of 0.9 and an α (significance level) of 0.01. The loss ratio of follow-up was 10%. Approximately 220 to 260 cases were recruited in this study. We conducted a retrospective cohort study that included a consecutive series of patients with painful hips or knees after total joint arthroplasty (TJA) who underwent debridement antibiotic irrigation and implant retention (DAIR), one-stage or the first stage of a two-stage revision TKA or THA for acute or chronic PJI or revision TKA or THA for aseptic failures in our hospital between January 2017 and December 2019. This study was approved by the Ethical Committee of our institution and informed consent was obtained in all cases before surgery was performed. Acute PJI was defined if the infection had been present for less than 3 months. The presence of PJI for more than 3 months was defined as chronic PJI. Some patients were excluded due to a lack of laboratory test results (*n* = 3) and periprosthetic fracture (*n* = 24). The cohort included 44 patients with acute PJI, 69 patients with chronic PJI, and 152 patients with aseptic failures. To rule out some possible bias, venous thrombosis (*n* = 12), autoimmune diseases (*n* = 9), malignancies (*n* = 3), concurrent infections in other organs (*n* = 8), and recent use of antibiotics 2 weeks before (*n* = 12) were evaluated separately. Ultimately, 31 acute PJI patients were assigned to group A, 51 chronic PJI patients were assigned to group B, and 139 aseptic failure patients were assigned to group C (Fig. 1). The basic demographic data are shown in Table 1, and there was no significant difference in body mass index (BMI) between the PJI and aseptic failures. However, compared with aseptic failures, acute PJI patients’ male proportion and age were greater (*P <* 0.05). Additionally, the PJI patients’ knee revision rate was higher (*P <* 0.05).

**Table 1:** Demographics of the groups

	Group A (=31)	Group B (*n* = 51)	Group C (*n* = 139)	*P* Valuet (Group A vs. C)	*P* Value (Group B vs. C)
Sex ^a^				0.043*	0.453
Male	17 (54.8%)	21 (41.2%)	49 (35.3%)		
Female	14 (45.2%)	30 (58.8%)	90 (64.7%)		
Age(y) ^b^	68 (60–74)	68 (53–74)	63 (49–71)	0.031*	0.093
BMI (kg/m^2^) ^c^	25.26 ± 3.55	25.22 ± 3.52	25.49 ± 4.12	0.768	0.679
Involved Joint ^a^				0.043*	0.024*
Knee	15 (48.4%)	24 (47.1%)	41 (29.5%)		
Hip	16 (51.6%)	27 (52.9%)	98 (70.5%)		

Group A, acute PJI group; Group B, chronic PJI group; Group C, aseptic failure group

^a^ The values were given as number of cases and the percentage in parentheses

^b^ Age was given as median with its interquartile range (25th - 75th) in parentheses

^c^ BMI were expressed as mean ± SD. BMI, body mass index; SD, standard deviation

* *P* < 0.05

### Diagnostic protocol and data extraction

According to the institution’s protocol, all patients who needed revision TKA or THA were well documented for their demographics, symptoms, signs, recent medicine use, and comorbidities in the electronic medical records system. Venous blood samples were collected by the nurses three to 7 days before surgery and were sent to the clinical laboratory within 2 h. These blood samples were used to test routine blood examinations (including neutrophil count, lymphocyte count, platelet count, mean platelet volume), CRP, ESR, liver function tests (including ALB, GLB, AGR), and coagulation examinations (including D-dimer, fibrinogen, FDP). Joint aspiration was performed for the PJI suspicious patients, and synovial fluid was sent to test white blood cell (WBC) count and polymorphonuclear neutrophil percentage (PMN%); at the same time, some synovial fluid was sent for aerobic and anaerobic cultures for 7–14 days. Additionally, at least three intraoperative samples were collected for aerobic and anaerobic cultures for 7–14 days. Histological analysis, including counting neutrophils per high-power field in 5 high-power fields, was routinely performed in all revision TKA or THA patients. All patients were followed up at least for 1 year. The 2013 criteria of the Musculoskeletal Infection Society (2013 MSIS), which is the most popular criterion, was used as the reference standard for the diagnosis of PJI [32]. Aseptic failures were defined as aseptic loosening, instability, wear, stiffness, implant breakage, unexplained pain, or aseptic reasons other than PJI. They did not fail because of infection within at least 1 year. All patients were followed for at least 1 year. All these data were well documented in our electronic medical records system and were carefully reviewed. Data extractors, follow-up investigators, and analyzers were blinded to the diagnosis of all patients.

### Laboratory assessments

Routine blood examinations were performed on a Sysmex XE2100 hematology analyzer (Sysmex, Japan) in our hospital. CRP was measured through turbidimetric inhibition immunoassay (Beckman, America). ESR was measured using Westergren methods. Liver function tests were measured on a BACKMAN-LX 20 automatic biochemical analyzer (Backman, America). Coagulation was measured on a Sysmex CA-500 automatic blood coagulation analyzer (Sysmex, Japan).

### Statistical analysis

Categorical variables were shown as counts (percentages), and the frequency distribution between the two groups was compared with the chi-square test. The comparisons of fibrinogen, ALB, GLB, AGR, platelet count, PVR, and BMI between the groups were performed using the independent *t*-test. The comparisons of D-dimer, FDP, PLR, NLR, ESR, CRP, and age between the two groups were analyzed using the Mann-Whitney *U* test. ROC curves were plotted, and AUC was compared between different biomarkers. The optimal cutoff was determined by the Youden index. The sensitivity, specificity, positive predictive value (PPV), and negative predictive value (NPV) of each test were calculated. The AUC values were determined to be excellent (0.900–1.000), good (0.800–0.899), fair (0.700–0.799), poor (0.600–0.699), and having no discriminatory capacity (0.500–0.599) [13]. A biomarker was treated as a good marker if its AUC was greater than 0.8. Comparisons of good diagnostic biomarkers between hip and knee PJI patients were analyzed. For patients with possible bias, including patients with venous thrombosis, autoimmune diseases, malignancies, and other organic infections, the recent use of antibiotics was evaluated separately based on the cutoff values of good diagnostic biomarkers. Culture results were shown, and good diagnostic biomarkers between positive and negative culture patients were analyzed. Analyses of diagnostic accuracy were exploratory. Statistical analysis and graphing were performed using SPSS version 24 (IBM Inc., Armonk, NY, USA) and GraphPad Prism software (Version 6; GraphPad Software Inc., San Diego, CA, USA). Statistical significance was set at *P <* 0.05.

## Results

### Biomarker results

**Fig. 2:**
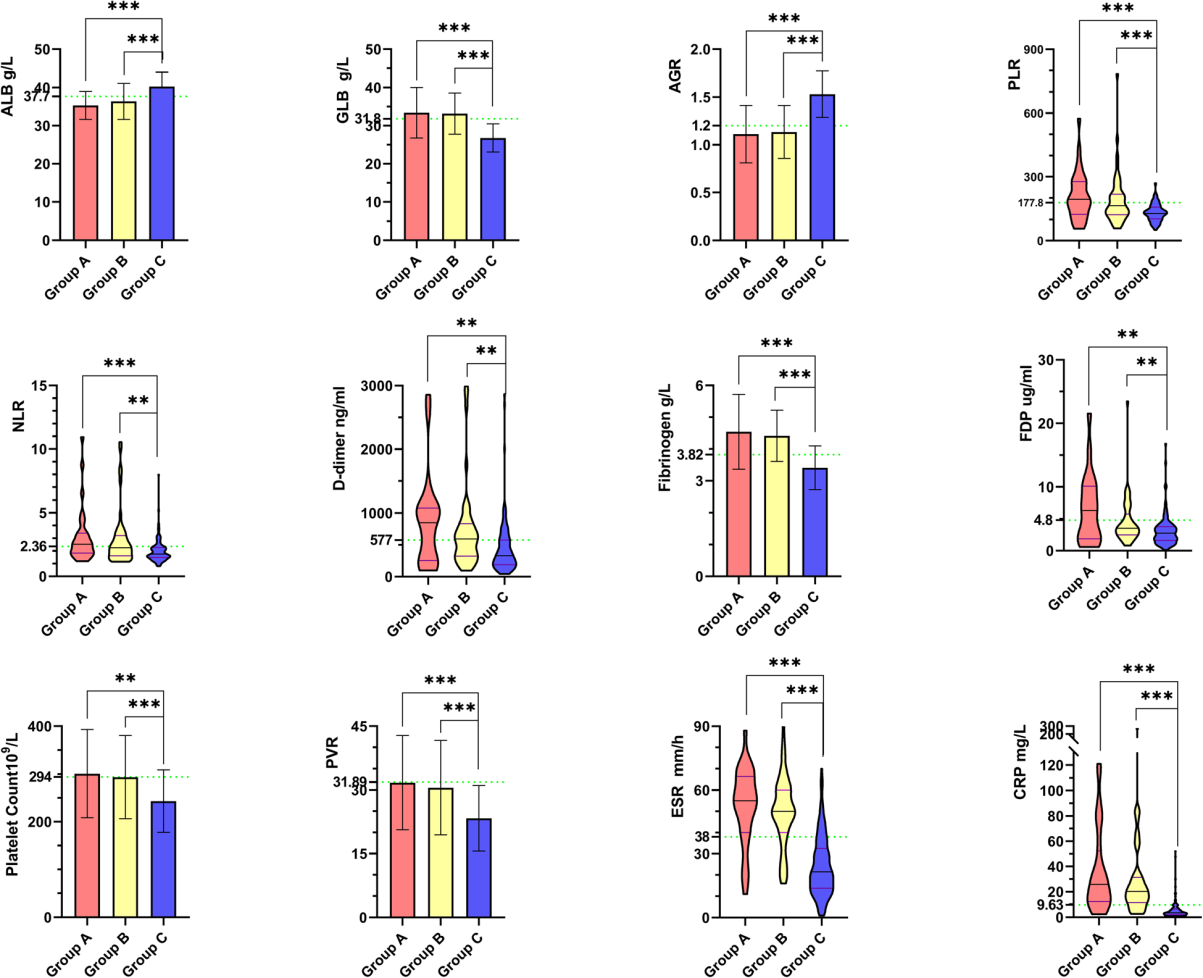
The difference of ALB, GLB, AGR, fibrinogen, D-dimer, FDP, platelet count, PVR, PLR, NLR, ESR and CRP levels in acute PJI group, chronic PJI group and aseptic failure group. Group A, acute PJI group; Group B, chronic PJI group; Group C, aseptic failure group. PJI, periprosthetic joint infection; ALB, albumin; GLB, globulin; AGR, albumin-to-globulin ratio; PLR, platelet-to-lymphocyte ratio; NLR, neutrophil-to-lymphocyte ratio; FDP, fibrin degradation product; PVR, platelet count to mean platelet volume ratio; ESR, erythrocyte sedimentation rate; CRP, C-reactive protein. The data of ALB, GLB, AGR, fibrinogen, platelet count and PVR were shown as mean with standard deviation (SD), PLR, NLR, D-dimer, FDP, ESR and CRP were shown as median with interquartile range (25th–75th). The dashed line indicates the optimal cutoff values. ** *P*<0.01; ***P<0.001

Compared with Group C, Groups A and B had significantly higher GLB, PLR, NLR, D-dimer, fibrinogen, FDP, platelet count, PVR, ESR, and CRP levels (*P <* 0.01; Table 2; Fig. 2). Nonetheless, the ALB and AGR levels of Groups A and B were significantly lower than those of Group C (*P* < 0.01). These biomarkers were not significantly different between Groups A and B (*P >* 0.05).

**Table 2:** Comparison of different biomarkers between PJI and aseptic groups

Variables	Normal Rang	Group A (*n* = 31)	Group B (*n* = 51)	Group C (*n* = 139)	*P* Value (A vs. B)	*P* Value (A vs. C)	*P* Value (B vs. C)
ALB^a^ (g/L)	40–55	35.33 ± 3.67	36.38 ± 4.75	40.29 ± 3.72	0.308	< 0.001	< 0.001
GLB^a^ (g/L)	20–40	33.41 ± 6.62	33.16 ± 5.40	26.78 ± 3.69	0.858	< 0.001	< 0.001
AGR^a^	1.2–2.4	1.11 ± 0.30	1.13 ± 0.28	1.53 ± 0.24	0.731	< 0.001	< 0.001
PLR^b^	–	194.12 (124.03–277.24)	163.93 (122.27–218.24)	128.69 (103.49–157.34)	0.308	< 0.001	< 0.001
NLR^b^	–	2.53 (1.83–3.40)	2.26 (1.62–3.21)	1.75 (1.49–2.28)	0.387	< 0.001	0.001
D-dimer^b^ (ng/ml)	<280	849 (256.5–1078.5)	592.5 (322.5–833.75)	331 (189.5–576)	0.344	0.003	0.001
Fibrinogen^a^ (g/L)	2–4	4.54 ± 1.18	4.41 ± 0.81	3.41 ± 0.69	0.572	< 0.001	< 0.001
FDP^b^ (ug/ml)	0–5	6.35 (1.86–10.11)	3.55 (2.48–5.78)	2.75 (1.6–3.8)	0.273	0.007	0.002
Platelet count^a^	125–350	300.74 ± 92.34	293.55 ± 86.91	243.22 ± 65.33	0.724	0.002	< 0.001
PVR^a^	–	31.73 ± 11.07	30.54 ± 11.09	23.35 ± 7.73	0.639	< 0.001	< 0.001
ESR (mm/h)^b^	0–15	55.00 (40.00–66.50)	50.00 (40.00–60.00)	21.50 (13.75–32.50)	0.332	< 0.001	< 0.001
CRP (mg/L)^b^	0–8	25.75 (12.28–52.28)	20.30 (11.50–31.40)	3.59 (2.23–6.31)	0.371	< 0.001	< 0.001

Group A, acute PJI group; Group B, chronic PJI group; Group C, aseptic failure group

^a^ The values were given as mean ± SD. SD, standard deviation

^b^ The values were given as median with its interquartile range (25th - 75th) in parentheses

*PJI* periprosthetic joint infection; *ALB* albumin; *GLB* globulin; *AGR* albumin-to-globulin ratio; *PLR* platelet-to-lymphocyte ratio; *NLR* neutrophil-to-lymphocyte ratio; *FDP* fibrin degradation product; *PVR* platelet count to mean platelet volume ratio; *ESR* erythrocyte sedimentation rate; *CRP* C-reactive protein

**Fig. 3:**
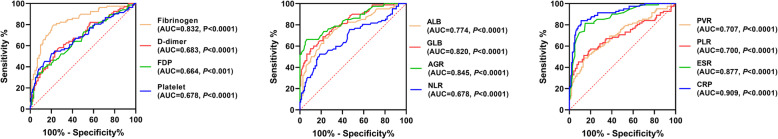
The ROC curves and AUCs of ALB, GLB, AGR, fibrinogen, D-dimer, FDP, platelet count, PVR, PLR, NLR, ESR and CRP. ROC, receiver operating characteristic curve; AUC, area under the curve; ALB, albumin; GLB, globulin; AGR, albumin-to-globulin ratio; PLR, platelet-to-lymphocyte ratio; NLR, neutrophil-to-lymphocyte ratio; FDP, fibrin degradation product; PVR, platelet count to mean platelet volume ratio; ESR, erythrocyte sedimentation rate; CRP, C-reactive protein

Table 3 shows the AUCs, Youden index values, optimal cutoff values, and PJI predictive values (sensitivity, specificity, PPV, NPV) of the twelve biomarkers. The ROC curve (Fig. 3) analysis showed that the diagnostic performance of CRP was excellent (AUC = 0.909), and that those of ESR (AUC = 0.877), AGR (AUC = 0.845), fibrinogen (AUC = 0.832) and GLB (AUC = 0.820) were good. The optimal cutoff value was based on the maximum Youden index. At the optimal cutoff value, the sensitivity, specificity, PPV and NPV were 83.95, 88.89, 81.93, 90.23% for CRP; 81.48, 85.07, 76.74, 88.37% for ESR; 66.25%, 93.48, 85.48, 82.69% for AGR; 78.48, 78.95, 68.89, 86.07% for fibrinogen; and 57.50, 89.86, 76.67, 78.48% for GLB, respectively. However, other biomarkers’ diagnostic performance levels were fair or poor, including ALB, PLR, PVR, NLR, D-dimer, FDP, and platelet count.

**Table 3:** Diagnostic performance of ALB, GLB, AGR, PLR, NLR, D-dimer, fibrinogen, FDP, platelet count, PVR, ESR and CRP for PJI

	AUC (95% CI)	Youden Index (95% CI)	Optimal Cutoff (95% CI)	Sensitivity(%) (95% CI)	Specificity(%) (95% CI)	PPV(%) (95% CI)	NPV(%) (95% CI)
ALB	0.774 (0.713–0.828)	0.450 (0.315–0.541)	37.7 (35.9–39.5)	67.50 (56.00–77.30)	77.54 (69.49–84.01)	63.53 (52.32–73.50)	80.45 (72.49–86.61)
GLB	0.820 (0.763–0.869)	0.474 (0.361–0.542)	31.8 (29.4–34.1)	57.50 (45.95–68.32)	89.86 (83.26–94.14)	76.67 (63.66–86.22)	78.48 (71.10–84.44)
AGR	0.845 (0.790–0.891)	0.597 (0.468–0.690)	1.2 (1.16–1.46)	66.25 (54.72–76.21)	93.48 (87.62–96.78)	85.48 (73.72–92.75)	82.69 (75.64–88.09)
PLR	0.700 (0.635–0.760)	0.376 (0.242–0.465)	177.8 (146.8–214.1)	51.22 (40.01–62.32)	80.58 (72.82–86.60)	60.87 (48.35–72.17)	73.68 (65.81–80.33)
NLR	0.678 (0.612–0.739)	0.337 (0.211–0.444)	2.36 (1.77–2.43)	52.44 (41.18–63.47)	81.30 (73.61–87.21)	62.32 (49.80–73.46)	74.34 (66.51–80.92)
D-dimer	0.683 (0.612–0.749)	0.311 (0.160–0.427)	577 (433–953)	55.22 (42.64–67.21)	75.83 (67.00–82.97)	56.06 (43.35–68.07)	75.21 (66.38–82.40)
Fibrinogen	0.832 (0.775–0.880)	0.574 (0.446–0.665)	3.82 (3.75–4.27)	78.48 (67.52–86.62)	78.95 (70.85–85.34)	68.89 (58.14–78.00)	86.07 (78.35–91.44)
FDP	0.664 (0.592–0.732)	0.271 (0.132–0.362)	4.8 (2.3–6.5)	38.81 (27.38–51.52)	88.33 (80.88–93.24)	65.00 (48.26–78.90)	72.11 (64.01–79.03)
Platelet Count	0.678 (0.612–0.739)	0.323 (0.198–0.414)	294 (215.6–333.0)	52.44 (41.18–63.48)	79.86 (72.04–85.99)	60.56 (48.24–71.74)	74.00 (66.09–80.65)
PVR	0.707 (0.642–0.766)	0.351 (0.230–0.453)	31.89 (27.37–40.71)	48.78 (37.68–59.99)	86.33 (79.23–91.36)	67.80 (54.24–79.03)	74.07 (66.50–80.49)
ESR	0.877 (0.825–0.917)	0.866 (0.547–0.752)	38 (34–44)	81.48 (70.98–88.93)	85.07 (77.65–90.43)	76.74 (66.16–84.89)	88.37 (81.25–93.13)
CRP	0.909 (0.862–0.944)	0.728 (0.622–0.810)	9.63 (9.35–14)	83.95 (73.75–90.85)	88.89 (82.04–93.44)	81.93 (71.63–89.21)	90.23 (83.55–94.48)

*AUC* area under the curve; *CI* confidence interval; *PPV* positive predictive value; *NPV* negative predictive value; *PJI* periprosthetic joint infection; *ALB* albumin; *GLB* globulin; *AGR* albumin-to-globulin ratio; *PLR* platelet-to-lymphocyte ratio; *NLR* neutrophil-to-lymphocyte ratio; *FDP* fibrin degradation product; *PVR* platelet count to mean platelet volume ratio; *ESR* erythrocyte sedimentation rate; *CRP* C-reactive protein

### Different joint results

Table 4 shows the comparison between hip (*n* = 43) and knee (*n* = 39) PJI. There was no significant difference between hip and knee PJI in GLB (32.56 vs. 34.06, *P >* 0.05), AGR (1.16 vs. 1.09, *P >* 0.05), fibrinogen (4.37 vs. 4.56, *P >* 0.05), ESR (50.0 vs. 54.0, *P* > 0.05), and CRP (20.3 vs. 22.9, *P* > 0.05). Based on the optimal threshold, the diagnostic accuracies of GLB, AGR, fibrinogen, ESR, and CRP were 55.81, 67.44, 81.40, 81.40, 86.05%, respectively, in hip PJI, while the diagnostic accuracies of GLB, AGR, fibrinogen, ESR, and CRP were 61.54, 66.67, 79.49, 79.49, 79.49%, respectively, in knee PJI.

**Table 4:** Comparison of GLB, AGR, fibrinogen, ESR and CRP between hip and knee PJI

Variables	Normal Rang	Hip PJI (*n* = 43)	Knee PJI (*n* = 39)	Statistics Test	*P* Value
GLB (g/L)^a^	20–40	32.56 ± 5.52	34.06 ± 6.15	*t* = −1.152	0.253
AGR^a^	1.2–2.4	1.16 ± 0.28	1.09 ± 0.29	*t* = 0.973	0.333
Fibrinogen (g/L)^a^	2–4	4.37 ± 0.80	4.56 ± 1.10	*t* = 0.870	0.387
ESR (mm/h)^b^	0–15	50.0 (40.0–60.0)	54.0 (40.0–62.5)	*Z =* -0.526	0.599
CRP (mg/L)^b^	0–8	20.3 (11.2–30.9)	22.9 (12.6–56.9)	*Z =* -0.804	0.421

*GLB* globulin; *AGR* albumin-to-globulin ratio; *ESR* erythrocyte sedimentation rate; *CRP* C-reactive protein

^a^ The values were given as mean ± SD. SD, standard deviation

^b^ The values were given as median with its interquartile range (25th - 75th) in parentheses

### Comorbidities analysis

In particular, we evaluated the diagnostic performance of GLB, AGR, fibrinogen, ESR, and CRP in patients with venous thrombosis, autoimmune diseases, malignancies, and concurrent infections in other organs based on the optimal threshold (Table 5). The diagnostic accuracies of ALB, AGR, fibrinogen, and ESR were nearly identical (75%) and lower than that of CRP (91.67%) in patients with venous thrombosis. The diagnostic accuracies of fibrinogen and CRP were the same (77.78%) and outstanding compared to others in patients with combined autoimmune diseases. The diagnostic accuracies of GLB and fibrinogen were equal to (100%) and better than those of other markers (66.67%) in patients with combined malignancies. Nevertheless, for patients with concurrent infections in other organs, the accuracies of GLB, AGR, and CRP all achieved 100%.

**Table 5:** Diagnostic performance of GLB, AGR, fibrinogen, ESR and CRP for patients with some comorbidities

Comorbidities and Numbers	GLB		AGR		Fibrinogen		ESR		CRP	
	PJI	Aseptic	PJI	Aseptic	PJI	Aseptic	PJI	Aseptic	PJI	Aseptic
Venous Thrombosis (*n* = 12)	4 (2)	8 (7)	4 (2)	8 (7)	4 (1)	8 (7)	4 (2)	8 (7)	4 (3)	8 (8)
Autoimmune Diseases(*n* = 9)	6 (2)	3 (3)	6 (3)	3 (3)	6 (5)	3 (2)	6 (3)	3 (2)	6 (4)	3 (3)
Malignancies (*n* = 3)	1 (1)	2 (2)	1 (1)	2 (1)	1 (1)	2 (2)	1 (1)	2 (1)	1 (1)	2 (1)
Concurrent Infections(*n* = 8)	8 (7)	0	8 (7)	0	8 (6)	0	8 (6)	0	8 (7)	0

*GLB* globulin; *AGR* albumin-to-globulin ratio; *ESR* erythrocyte sedimentation rate; *CRP* C-reactive protein

### Recent use of antibiotics results

Table 6 shows the comparison between PJI patients (*n* = 12) who used antibiotics 2 weeks before and PJI patients (*n* = 82) who had not used antibiotics recently. There were no significant differences between the cases of PJI in patients who had recently used and had not used antibiotics in terms of GLB (33.87 vs. 33.25, *P* > 0.05), AGR (1.20 vs. 1.13, *P* > 0.05), fibrinogen (4.45 vs. 4.46, *P* > 0.05), ESR (50.0 vs. 52.0, *P* > 0.05), and CRP (36.3 vs. 21.3, *P* > 0.05).

**Table 6:** Comparison of GLB, AGR, fibrinogen, ESR and CRP between the PJI patients who used antibiotics two weeks before and PJI patients who had not used antibiotics recently

Variables	Normal Rang	PJI Recent Use of Antibiotics (*n* = 12)	PJI No Use of Antibiotics (*n* = 82)	Statistics Test	*P* Value
GLB (g/L)^a^	20–40	33.87 ± 10.62	33.25 ± 5.83	*t* = 0.197	0.847
AGR^a^	1.2–2.4	1.20 ± 0.58	1.13 ± 0.28	*t* = 0.451	0.660
Fibrinogen (g/L)^a^	2–4	4.45 ± 1.16	4.46 ± 0.95	*t* = −0.019	0.985
ESR (mm/h)^b^	0–15	50.0 (37.8–58.5)	52.0 (40.0–61.5)	*Z =* -0.459	0.646
CRP (mg/L)^b^	0–8	36.3 (7.7–49.0)	21.3 (12.3–35.3)	Z = -0.453	0.651

*GLB* globulin; *AGR* albumin-to-globulin ratio; *ESR* erythrocyte sedimentation rate; *CRP* C-reactive protein

^a^ The values were given as mean ± SD. SD, standard deviation

^b^ The values were given as median with its interquartile range (25th - 75th) in parentheses

### Culture results

Table 7 shows the culture results of PJI patients. Among the 82 PJI patients, 59 achieved positive cultures (71.95%), and the two most common pathogens were *Staphylococcus epidermidis* (33.90%, 20 of 59) and *Staphylococcus aureus* (18.64%, 11 of 59). The positive culture rate (66.67%, 8 of 12) in the twelve PJI patients with antibiotics recently was lower than that in PJI patients not treated with antibiotics (71.95%, 59 of 82). There were no significant differences between the culture-positive and culture-negative PJI patients in terms of GLB (33.97 vs. 31.23, *P >* 0.05), AGR (1.09 vs. 1.22, *P >* 0.05), fibrinogen (4.52 vs. 4.30, *P >* 0.05), and CRP (21.7 vs. 20.3, *P* > 0.05) (Table 8). The ESR of positive culture PJI was significantly higher than that of negative culture PJI (54.0 vs. 46.0, *P* < 0.05). Based on the optimal threshold, the diagnostic accuracies of GLB, AGR, fibrinogen, ESR, and CRP were 62.71, 71.19, 83.05, 83.05, and 89.83%, respectively, in culture-positive PJI and 47.83, 56.52, 73.91, 73.91, and 65.22%, respectively, in culture-negative PJI.

**Table 7:** Culture results of PJI patients(*n* = 82)

Culture Results	Number of patients
Positive	59
*Staphylococcus epidemidis*	20
*Staphylococcus aureus*	11
*Staphylococcus capitis*	3
*Brucella*	2
*Pseudomonas aeruginosa*	2
*Enterobacter cloacae*	2
*Streptococcus mitis*	2
*Streptococcus sanguis*	2
*Proteus mirabilis*	1
*Bacillus subtilis*	1
*Bacillus firmus*	1
*Human Staphylococcus*	1
*Staphylococcus lugdunensis*	1
*Staphylococcus haemolyticus*	1
*Canidia albicans*	1
*Staphylococcus saprophyticus*	1
*Escherichia coli*	1
Polymicrobial	6
Negative	23

**Table 8:** Comparison of GLB, AGR, fibrinogen, ESR, and CRP between culture-positive and culture-negative PJI

Variables	Normal Rang	Culture-Positive PJI (*n* = 37)	Culture-Negative PJI (*n* = 14)	Statistics Test	*P* Value
GLB (g/L)^a^	20–40	33.97 ± 5.53	31.23 ± 6.32	*t* = 1.881	0.064
AGR^a^	1.2–2.4	1.09 ± 0.26	1.22 ± 0.33	*t* = −1.614	0.117
Fibrinogen (g/L)^a^	2–4	4.52 ± 1.02	4.30 ± 0.73	*t* = 0.878	0.382
ESR (mm/h)^b^	0–15	54.0 (46.0–64.3)	46.0 (26.0–54.0)	*Z = -*2.371	0.018
CRP (mg/L)^b^	0–8	21.7 (13.9–35.0)	20.3 (6.5–49.2)	*Z =* -1.330	0.183

*GLB* globulin; *AGR* albumin-to-globulin ratio; *ESR* erythrocyte sedimentation rate; *CRP* C-reactive protein

^a^ The values were given as mean ± SD. SD, standard deviation

^b^ The values were given as median with its interquartile range (25th - 75th) in parentheses

## Discussion

Based on a literature review, this study was the first to evaluate the diagnostic performances of ALB, GLB, and AGR in differentiating PJI and aseptic failure. We compared ALB, GLB, and AGR with some inflammatory biomarkers (D-dimer, fibrinogen, FDP, platelet count, PVR, PLR, NLR, ESR, and CRP). We found that GLB, AGR, fibrinogen, ESR, and CRP had good performance in the diagnosis of PJI. At the optimal cutoff point of AGR, apart from its low sensitivity (66.25%), the specificity (93.48%) was the highest among these biomarkers, reducing the misdiagnosis rate of PJI. Additionally, the PPV of AGR (85.48%) was the highest; that is, AGR was superior in predicting the diagnosis of PJI. The diagnostic value of fibrinogen was slightly lower than that of AGR based on the AUC analysis. However, the diagnostic value of ALB was fair, with an AUC, sensitivity, and specificity of 0.774, 67.50, and 77.54%, respectively. In terms of this study, GLB, and AGR were good biomarkers; they were two essential markers in liver function tests that were routinely performed before surgery with no time wasted and no further cost. Hence, GLB and AGR are two promising and adequate biomarkers to aid in diagnosing PJI.

Albumin is typically utilized to evaluate nutritional status; recent studies have demonstrated that albumin is a negative phase reactant, as its synthesis decreases during inflammation [33–35]. Previous research conducted by Miraeidi M et al. [22] found that inflammation could reduce albumin levels irrespective of the patients’ nutritional state. One possible explanation of this phenomenon was that inflammatory mediators could promote albumin escape from blood capillaries and reprioritize hepatic protein synthesis in favor of acute-phase reactants [36]. Globulin consists of many proteins associated with inflammation, such as complements, interleukin-6, and immunoglobulins; thus, elevated globulin reflects an inflammatory state [20, 37, 38]. As both decreased albumin and increased globulin played essential roles in inflammation, the AGR, which took albumin and globulin into account concurrently, could indicate the body’s inflammatory state more accurately [20, 26, 39, 40].

Coagulation-associated biomarkers are usually used to monitor venous thromboembolism and assess coagulation status before surgery. Recently, many studies have demonstrated that inflammation is closely related to the coagulation cascade [41, 42]. D-dimer, fibrinogen, and FDP were promising biomarkers for the diagnosis of PJI in some studies [8, 27]. In this study, fibrinogen’s diagnostic performance was good, and the results of sensitivity, specificity, and AUC were similar to Li et al.’s study [43]. However, the diagnostic values of D-dimer and FDP were limited, and this result was proven by Xu H et al.’s study [44]. In the clinic, fibrinogen could be used for diagnosis in patients suspected of PJI.

Gaertner F et al. [45] demonstrated the anti-infection role of platelets in collecting and bundling bacterial functions. Parvizi et al. [18] showed that PVR increased in PJI patients, and its diagnostic sensitivity, specificity, and AUC were 48.10, 80.85%, and 0.69, respectively, similar to the findings of this study. Compared with GLB, AGR, and fibrinogen, the diagnostic value of platelet count and PVR was limited.

PLR and NLR are simple biomarkers of inflammation. Previous research conducted by Zhao Z et al. [29] found that PLR and NLR were associated with chronic hepatitis B virus infection. In addition, Gasparyan A. Y. et al. [30] found that PLR could be an inflammatory marker in rheumatic diseases. In this study, we found that the diagnostic performance of PLR and NLR was limited, as the AUC, sensitivity and specificity of PLR were 0.700, 51.22, 80.58%, respectively, and those of NLR were 0.678, 52.44 and 81.30%, respectively.

In the analyses of different joints, we found that the GLB, AGR, fibrinogen, ESR, and CRP levels between hip and knee PJI patients were nearly the same. Furthermore, the diagnostic accuracies of these biomarkers were not largely different.

We evaluated the diagnostic accuracies of GLB, AGR, fibrinogen, ESR, and CRP in patients with comorbidities (venous thrombosis, autoimmune diseases, malignancies, and concurrent infections in other organs) separately and found that their diagnostic accuracies were good. As the number of cases was small, the diagnostic accuracies of these biomarkers should be evaluated in a larger number of cases in the future.

The recent use of antibiotics might influence inflammation biomarkers. However, in this study, we found no significant difference between PJI patients who recently used and did not recently use antibiotics in GLB, AGR, fibrinogen, ESR, and CRP. Therefore, the use of antibiotics had little influence on these biomarkers, and it was attractive to use these three biomarkers in the PJI diagnosis of patients who had recently used antibiotics.

Benito N et al. [46] conducted a multicenter cohort study of PJI and obtained 2288 cases with the microbiologic diagnosis. In their research, *Staphylococcus aureus*, *Staphylococcus epidermidis*, *Escherichia coli*, *Pseudomonas aeruginosa*, *Enterococcus faecalis*, and *Propionibacterium acnes*, in decreasing order, were involved in more than 80% of PJIs. In our study, *Staphylococcus epidermidis* was the most common pathogen (33.90%, 20 of 59), followed by *Staphylococcus aureus* (18.64%, 11 of 59). This difference was caused by the bacterial spectrum varying among different areas. There was no significant difference between the culture-positive and culture-negative PJI patients in GLB, AGR, fibrinogen, and CRP. However, the diagnostic accuracies of these biomarkers in culture-negative PJI were lower than those of culture-positive PJI: GLB (47.83% vs. 62.71%), AGR (56.52% vs. 71.19%), fibrinogen (73.91% vs. 83.05%), and CRP (65.22% vs. 89.83%). Therefore, more attention is needed in the diagnosis of culture-negative PJI. The positive culture rate of PJI patients with recent use of antibiotics was lower than that of those who had not recently used antibiotics; thus, the use of antibiotics decreased the culture rate.

There are some limitations to this study. The most important limitation related to this study is the retrospective nature. Another limitation is the small sample size of this study, necessitating further large-scale, prospective, and multicenter studies. Finally, this study lacks validation, which may lead to the reduced reliability of these new biomarkers; we will verify this hypothesis in future studies.

## Conclusion

Our study demonstrated that GLB, AGR, and fibrinogen were promising biomarkers in the diagnosis of PJI.

## Data Availability

The data used and analyzed during the current study are available from the corresponding author on reasonable request.

## Abbreviations

BMI: body mass index
MSIS: Musculoskeletal infection society
PJI: Periprosthetic joint infection
THA: Total hip arthroplasty
TKA: Total knee arthroplasty
TJA: Total joint arthroplasty
DAIR: Debridement antibiotic irrigation and implant retention
ALB: Albumin
GLB: globulin
AGR: Albumin-to-globulin ratio
PLR: Platelet-to-lymphocyte ratio
NLR: Neutrophil-to-lymphocyte ratio
FDP: Fibrin degradation product
PVR: Platelet count to mean platelet volume ratio
ESR: Erythrocyte sedimentation rate
CRP: C-Reactive protein
WBC: White blood cell
PMN: Polymorphonuclear neutrophil
SD: Standard deviation
ROC: Receiver operating characteristic
AUC: Area under the curve
CI: Confidence interval
PPV: Positive predictive value
NPV: Negative predictive value
